# A Survey Study on Clinicians’ Rationale and Attitude Towards the Prescription of Antipsychotic Polypharmacy in the East Perth Metropolitan Area in Western Australia

**DOI:** 10.7759/cureus.37234

**Published:** 2023-04-07

**Authors:** Suzie Uloma Ajayi, Praveena Arora

**Affiliations:** 1 Research and Development, Oceania University of Medicine, Apia, WSM; 2 Older Adult Mental Health Service, Royal Perth Bentley Health Service, Perth, AUS

**Keywords:** prescribing practice, prescribers attitude, prescription of antipsychotic polypharmacy, clinicians prescribing rationale, antipsychotic polypharmacy (apps)

## Abstract

Objective

Patients challenging refractory and residual psychotic symptoms have led to the concomitant use of combined antipsychotics, which was later introduced and labelled ‘antipsychotic polypharmacy’ (APP). Many clinicians have become somewhat hesitant to adjust psychotropic medication dosages, resulting in a higher dose and combination prescription of antipsychotics and only achieving modest success. This study examines and investigates clinician perspectives and the rationale for the prescription of antipsychotic polypharmacy.

Methods

A structured questionnaire designed to reflect 15 target-directed questions evaluating clinicians’ attitudes and rationale on antipsychotic polypharmacy prescription was administered from November to December 2022. Information was obtained from inpatient and outpatient prescribers (psychiatric consultants) in two government-funded psychiatric facilities and outpatient clinics in the East Perth Metropolitan Area in Western Australia.

Results

After exclusion, a total of 45 participants’ responses were analysed. These results suggest a higher frequency of questions relating to the prescription of APP based on previous prescribers’ consultation and recommendation from a prior treating team; senior nurses’ pressure impacting clinicians’ decisions on APP perception; and the patient’s risk of aggression impacting the clinician’s rationale for the prescription of APP.

Conclusions

Clinicians’ rationale and attitude towards the prescription of APP are mostly influenced by recommendations from prior treatment teams or consultations and patients’ risk for aggression without compromising practice guidelines. Our findings also highlight the need to evaluate prescribers’ attitudes and how it presents an opportunity to enhance patients’ holistic outcomes.

## Introduction

Antipsychotic medication remains the main pharmacological agent in treating psychiatric illnesses, especially schizophrenia [1,2], which is considered a chronically disabling condition. Its management requires immense input from clinicians and family members, not to mention the financial burden it places on the health system. This is mostly due to the patient’s fluctuating presentation, medication noncompliance, and inadvertently deteriorating clinical sequence, amongst many other things [3]. Antipsychotic monotherapy (APM) was officially recommended by reputable treatment guidelines, randomised cohort studies, and well-designed clinical trial evidence as the gold-standard therapy in routine psychiatry practices [4]. However, in the real clinical setting, APM remains a substantial challenge despite several pharmacologic innovations, and its efficacy is not as satisfactory or as yielding, especially in treatment-resistant cohorts [5,6]. This, therefore, justifies the use of a second treatment option, antipsychotic polypharmacy (APP). Despite the weak and controversial evidence supporting the use of APP, it is clinically recommended as the cornerstone for successful outcomes, especially in the acute psychotic phase, prevention of relapse, and addressing antidopaminergic activities in patients in the acute mental health setting [6].

Understanding the clinicians’ justification for introducing and maintaining APP and prescribing preferences continues to be the focus of numerous studies and scientific reports. Additionally, as the cost of drugs continues to soar due to the increasing cost of healthcare around the world [7, 8], antipsychotic polypharmacy remains a questionable approach. Interestingly, among the top 10 identified underlying reasons for APP-prescribing culture, individualised prescribing decisions, ambiguous local treatment guidelines, and algorithmic imprecision were mostly highlighted [9,10]. A study conducted in the United States identified the increasing accessibility of psychotropic medication, especially in the "off-label" context, as a pertinent rationale for prescribers’ choice [2]. The prevalence of APP and clinicians’ attitudes have been poorly reported in prior studies, largely due to publication biases on the effectiveness and safety of APP [2,7]. According to Correll et al., in a recent survey conducted in a psychiatric teaching hospital in the US, prescribers who had more clinical experience in the acute mental health setting were often less bothered about APP prescription and therefore favoured its use [7]. Another study was a Japanese survey conducted in Japan in 2013, highlighting prescribers’ attitudes towards APP; however, no significant outcome data were reported to support clinicians' prescribing habits [11]. Similarly, potential advocates for APP posit that the so-called "rational polypharmacy", somewhat justifies the use of APP in certain situations where a "crossover" approach is inevitable as patients switch from one medication to another and require an overlapping medication regimen [1,12].

Approximately two decades ago, APP was labelled "a legitimate but unnecessary use of psychotropic agents" [13], with the reported negative associations to QTc prolongation, extrapyramidal symptoms, cognitive dysfunction, potential drug interactions, poor medication adherence, increased financial burden (personal, family, and public health), and increased risk of metabolic syndrome [6]. Interestingly, despite the highlighted side effects, recent studies continue to indicate that APP continues to enjoy enormous patronage from clinicians [2,13]. APP has been shown to reduce patients’ positive symptoms regardless of the disease phase and chronicity with minimal consequences [4]. It is therefore reasonable to state that APP should no longer be referred to as the "dirty little secret" [13]. This also highlights another relevant finding by Barbui et al., where the total combination therapy concurrently prescribed to a patient during their inpatient treatment was an important predictive factor that determined how much medication was also provided at discharge [14]. For instance, patients who were managed with three or more APP had more chances to be discharged with the same or more loading doses, thus increasing the possibility of subsequent excessive long-term dosing.

Understanding the disconnect between the perceived side effects of APP and what its continued use warrants are important. Therefore, to address this knowledge gap, this study seeks to examine clinicians’ rationale and attitude towards the perception of APP. We are unable to identify any previous study highlighting these attitudes among psychiatric providers in the East Perth Metropolitan Area of Western Australia.

## Materials and methods

Study location and setting

All questionnaires were obtained from all inpatient and outpatient clinicians in two government-funded psychiatric facilities and one outpatient clinic in the East Perth Metropolitan Area of Western Australia. For simplicity in explanation, we interchangeably use the term "clinicians", to refer to prescribers and consultants in the mental health setting. The survey was conducted between November and December 2022, and the data in this report were collected only from consultant psychiatrists. The ethical approval for this survey was granted by the Institutional Review Board of the Oceania University of Medicine, approval number 22-5510SA. This is a paper survey distributed by hand to the clinicians, with no incentive provided for participation.

Inclusion criteria

This was based on criteria that were relevant to the study to minimise bias and reflect a sufficient response. These include inpatient and outpatient clinicians (consultant psychiatrists), the prescribers’ years of clinical experience (five years and above), and the number of patients seen by the prescriber per week (at least 10 patients per week). All of these were sufficient to meet the inclusion criteria. We aimed to capture the opinions of psychiatrists with a certain level of experience. Furthermore, all antipsychotic polypharmacy (a first-generation antipsychotic and a second-generation antipsychotic medication) was regarded as "antipsychotic polypharmacy" in this context.

Exclusion criteria

Exclusion criteria were necessary to attain reliable data for this study. Therefore, psychiatric facilities outside the East Perth Metropolitan Area of Western Australia, visiting prescribers or locum tenens clinicians in these facilities, and interns (resident medical officers or duty medical officers) were all excluded as target participants.

Instrument

The questionnaire developed for this survey was inspired by the "Prescriber’s Reason for Antipsychotic Combination Treatment Questionnaire" (PRACT-Q) [2] (Appendices 1, 2) and shared similarities in the concept. This is a semi-structured questionnaire designed to assess several aspects of the topic. Prescriber information, such as years of practice experience and the total number of patients seen per week, was also included.

Data analyses

Descriptive statistical analysis (measures of frequency) was used to summarise and describe the clinicians’ responses. The proportion of clinicians who responded in a certain way to our structured questions is presented. A simple Microsoft Excel application was utilised to show the percentages for simplicity of understanding.

Ethical considerations

The questionnaire used for this project was created to ensure the anonymity of all participating clinicians. No identifying information was acquired, and participants were informed that their participation was voluntary, giving them the option to decline or opt out of the study. Each questionnaire was composed of two structured questions. Reading and completing the questionnaire was considered to imply consent from the participant.

## Results

Clinicians’ demographic characteristics

A total of 50 questionnaires were given out to the clinicians; 45 questionnaires were returned with all the questions completed, and the remaining five questionnaires had a few questions unanswered. Those five questionnaires were deemed incomplete and discarded. Of the 45 participants, the range in clinical experience was five to 14 years, with a mean of eight years. The number of patients seen by inpatient providers per week was approximately 25, while the number of patients seen by outpatient prescribers per week was approximately 33.

Main outcome

For simplicity, Table 1 below shows the distribution of responses to each question about clinicians’ rationale and attitude towards prescribing APP.

**Table 1:** Frequency in distribution and percentages of responses to questions about clinicians’ rationale and attitude towards the APP N=45 is the total number of participants

		N=45	%
Q1	How likely are you to prescribe APP by self-initiation?		
	Not at all likely	0	0%
	Unlikely	4	9%
	Neutral	4	9%
	Very likely	16	36%
	Extremely likely	21	47%
Q2	How likely are you to prescribe APP based on previous prescribers’ consultation/advice?		
	Not at all likely	3	7%
	Unlikely	4	9%
	Neutral	5	11%
	Very likely	21	47%
	Extremely likely	12	27%
Q3	How likely are you to reduce the dose of APP and combine a new antipsychotic medication?		
	Not at all likely	7	16%
	Unlikely	5	11%
	Neutral	11	24%
	Very likely	14	31%
	Extremely likely	8	18%
Q4	How likely are you to think that three-type combined medication is too much?		
	Not at all likely	16	36%
	Unlikely	11	24%
	Neutral	15	33%
	Very likely	0	0%
	Extremely likely	3	7%
Q5	How likely are you to continue to prescribe APP in your usual practice, if the patient presents with unwanted side effects?		
	Not at all likely	3	7%
	Unlikely	6	13%
	Neutral	21	47%
	Very likely	10	22%
	Extremely likely	5	11%
Q6	How likely are you to add a new antipsychotic medication in addition to the already existing regime?		
	Not at all likely	0	0%
	Unlikely	0	0%
	Neutral	8	18%
	Very likely	17	38%
	Extremely likely	20	44%
Q7	How likely are you to prescribe APP based on personal preference?		
	Not at all likely	4	9%
	Unlikely	6	13%
	Neutral	14	31%
	Very likely	13	29%
	Extremely likely	8	18%
Q8	How likely are you to prescribe APP for a patient for whom you have limited information i.e., a patient previously lost to follow-up?		
	Not at all likely	6	13%
	Unlikely	6	13%
	Neutral	12	27%
	Very likely	13	29%
	Extremely likely	8	18%
Q9	How likely are you to prescribe APP based on the recommendation from the prior treating team?		
	Not at all likely	1	2%
	Unlikely	3	7%
	Neutral	5	11%
	Very likely	20	44%
	Extremely likely	16	36%
Q10	How likely would overwhelming workload and senior nurses’ pressure impact your decision to prescribe APP?		
	Not at all likely	6	13%
	Unlikely	1	2%
	Neutral	4	9%
	Very likely	15	33%
	Extremely likely	19	42%

The results of research questionnaire #1 (Appendix 1) indicated that clinicians responded as "extremely likely" (47%) and "very likely" (36%), when asked how likely they were to prescribe an APP by self-initiation. Psychiatrists favour prescribing APP based on previous prescribers’ consultations or advice, with 47% rating it "very likely" and 27% "extremely likely". There were 24% "neutral" and 31% "extremely likely" responses to the question as to how likely clinicians would reduce the dose of APP and combine a new antipsychotic medication. Overall, clinicians felt "not at all likely" at 36% and "neutral" at 33% concerning how likely it is to think that three-type combined medication is too much. Another result was recorded on question five, with a 47% "neutral" score for how likely it is for prescribers to continue to prescribe APP in their usual practice if the patient presents with unwanted side effects.

On the other hand, question six reveals 44% as "extremely likely" and 0% as "not at all likely", when asked how likely clinicians are to add a new antipsychotic medication in addition to the already existing regime. Furthermore, prescribers revealed a 31% "neutral" score on the question of how likely psychiatrists are to prescribe APP based on personal preference. We recorded a balanced score on the question of how likely prescribers are to prescribe APP for a patient about whom you have limited information, for example, a patient who previously was lost to follow-up, with 18% for "not at all likely", 18% for "unlikely", and 18% for "extremely likely", respectively. Interestingly, there was a significant increase (44% "very likely" and 36% "extremely likely") in how likely clinicians are to prescribe APP based on the recommendation from the prior treating team. Lastly, there is an impressive score in question 10, on how likely overwhelming workload and senior nurses’ pressure would impact your decision to prescribe APP, with a score of 42% "extremely likely" and 33% "very likely".

Table 2, the second part of the questionnaire, captures the responses from participants with a "yes" or "no" response.

**Table 2:** The second part of the questionnaire with a "yes" or "no" response

		Yes	No
Q1	Is your prescription rationale linked to medication cross-titration, failed clozapine trial, and clozapine intolerance?	56%	44%
Q2	Do the local and national guidelines impact your prescription practice?	87%	13%
Q3	Does the patient’s risk for aggression impact your rationale for the prescription of an APP	64%	36%
Q4	Based on your expert opinion, does the justification for the prescription of APP correlate to its increased efficacy?	64%	36%

Research questionnaire #2 (Appendix 2) asked "yes" or "no" questions about whether certain situations influenced the clinicians’ prescription of APP. A total of 56% of participants responded "yes" and 44% responded "no" when asked if medication cross-titration, failed clozapine, and intolerance influence the prescription of APP. Secondly, when asked if local and national guidelines impact prescription practice, 87% responded ‘yes’ and 13% responded "no". Furthermore, 64% acknowledged that the patient’s risk of aggression impacts their rationale for the prescription of APP, and 36% marked "no". Lastly, 64% of clinicians agreed that the prescription of APP correlates to its increased efficacy, while 36% of the participants were not in support of the same.

## Discussion

A total of 45 clinicians were included in this study, all of whom were consultant psychiatrists with an average of eight years of clinical experience, and approximately 47% of these clinicians are extremely likely and 36% are likely to prescribe APP by self-initiation. Furthermore, 47% of our subjects’ responses favour the pattern of prescription based on previous prescribers’ consultations or advice. One potential explanation for this may stem from the fact that clinicians who inherit treatment-resistant patients are more likely to continue in the same treatment pattern as the previous treating team [9,15]. Similarly, these findings were also highlighted in a recent study by Khalid et al. [16], reporting that routine practice may be a reflection of current practices that junior doctors are exposed to during psychiatric training. Yet, another study also found that up to 75% of clinicians’ justification for polypharmacy prescription had been a passed-on practice from the previous treating team [2,9].

Furthermore, our study reveals that 49% of prescribers were very or extremely likely to reduce the dose of medication and combine a new antipsychotic, while 60% are of the opinion that a three-type combined antipsychotic medication is not too much. This may indicate some inconsistency in clinicians’ opinions versus actual practice. Research seems to conclude that most clinicians refer to the recommended guidelines and treatment algorithm and follow a step-wise approach in the introduction of the APP [4,3]. Although the guidelines accommodate some exemptions in certain clinical circumstances, the measure of clinicians’ compliance with these evidence-informed recommendations and deprescribing practices remains debatable and should warrant further studies in this area; therefore, our study considers this a potential limitation.

Our study on clinicians’ rationale and attitude showed an unexpected finding on prescribing APP for patients presenting with side effects. A neutral score of 47% could also indicate that there are additional factors that the clinician will consider in reaching such a decision. For instance, clinicians could rationalise the severity of the side effect against its frequency of occurrence. Significant data for harm is more compelling, with published evidence of clinical adverse effects associated with the co-prescription of antipsychotics [2,9,11], such as extrapyramidal side effects (EPSE), metabolic complications, sexual dysfunction, and cardiac arrhythmias [11,17]. Nevertheless, a proposal for the introduction of certain lifestyle modifications, such as regular exercise, routine electrocardiograms (ECGs), regular follow-up with a local general practitioner (GP), good sleep hygiene, and the prescription of medications to counter these associated side effects, is routinely recommended alongside. It is expected that the perceived side effects of APP would discourage prescribers' attitudes towards it. However, this could be subjective, making it an inaccurate approach to use in measuring clinicians’ attitudes.

Additionally, we also found interesting results on the effect of how an overwhelming workload and senior nurses’ pressure impact clinicians’ decisions to prescribe APP. Approximately 64% of clinicians agree that a patient’s risk for aggression impacts their rationale for the prescription of APP. In a study conducted in two acute care hospitals in Melbourne, it was demonstrated that nurses anticipating patients’ worsening hyperactive behaviours was a major concern [18]. Conversely, this reflects the subtle clinical judgement of experienced nurses that is then passed on to clinicians and further impacts a hard-line approach in the prescription of antipsychotic polypharmacy. Interestingly, this semi-structured interview also revealed that doctors were more concerned about the safety of the nurses, especially as they are the primary caregivers with the increased potential for aggression from challenging patients [18]. This implies that nurses contribute as primary decision-makers and influence doctors’ decisions in some regards. Nevertheless, staff safety and maintaining a safe working environment serve as key decision-making components in this context.

This study highlights the importance of rational polypharmacy, which primarily aims to address issues relating to the response to traditional antipsychotic monotherapy. It also adds to a developing body of knowledge on prescribers’ rationale and attitude towards APP. The questionnaire captures a good description of participants' perceptions and other influencing factors that shape their attitudes towards APP. Yet, it remains inconclusive in reflecting an actual clinical practice due to individual patients’ demographics, treatment variables, and intricate clinical circumstances. Another limitation of our study is that this research was limited to two psychiatric facilities in the East Perth Metropolitan Area; hence, the generalisation of the findings may have been skewed. Expanding our catchment area could potentially enhance the demography and boost reliability. Equally important is also the underlying impact of organisational culture on clinicians’ attitudes, which could also have a contributory effect. Consequently, recruiting participants from several facilities across different geographical locations can be considered a strategy to mitigate this in future studies. Lastly, it is noteworthy, however, to state that statistically relevant results may not necessarily correlate to any practical or clinical implication or significance, and therefore caution is required in the analysis of these findings. This is mostly due to the subjective nature of this study, as it is limited to measuring clinicians’ attitudes and not their behaviour.

## Conclusions

Our study reflects a remarkably positive attitude towards APP. Clinicians’ rationale can be attributed to a combination of institutional clinical guidelines and sound clinical reasoning. Their clinical judgement was best indicated by an outstanding record of clinicians’ prescription attitudes and rationale based on the recommendation of the prior treating team and the impact of the patient’s risk for aggression. Interestingly, this approach suggests clinicians are willing to endorse and maintain a treatment regime that has been tried and tolerated by the patient over time. This will invariably ensure consistency in the treatment management plan, with the aim of significantly reducing relapse and promoting medication compliance. This simply reveals the extent and effort that experienced clinicians have employed to formulate a useful strategy for enhancing the effectiveness of antipsychotic polypharmacy. Thus, clinicians' continued practice represents an opportunity to boost the outcome opportunities for treatment-resistant patients. Our study therefore may be considered a crucial initial step towards generating state-level data aimed at understanding the impacts of clinicians' prescribing practice in the East Perth Metropolitan Area of Western Australia. Consequently, if possible, future studies are required to explore more expansive clinicians’ behaviours in the actual clinical setting and not necessarily attitudes and other salient factors. High-priority, well-controlled research in this area is therefore crucial to significantly narrowing the gap between clinical practice and the best evidence.

## Appendices

Research questionnaires

Appendix One: Research Questionnaire #1

**Table 3:** Research questionnaire #1

	Scales 1= not at all likely; 2= unlikely; 3= Neutral ; 4= very likely 5 = extremely likely	1	2	3	4	5
1	How likely are you to prescribe an APP by self-initiation?					
2	How likely are you to prescribe APP based on previous prescribers’ consultation/advice?					
3	How likely are you to reduce the dose of medication and combine a new antipsychotic drug?					
4	How likely are you to think that 3-type combined medication is too much?					
5	How likely are you to continue to prescribe APP in your usual practice, if the patient presents with unwanted side effects?					
6	How likely are you to add a new antipsychotic medication in addition to the already existing regime?					
7	How likely are you to prescribe APP based on personal preference?					
8	How likely are you to prescribe APP for a patient for whom you have limited information, for example, a patient previously lost to follow-up?					
9	How likely are you to prescribe APP based on the recommendation from the prior treating team?					
10	How likely would overwhelming workload and senior nurses’ pressure impact your decision to prescribe APP?					

This questionnaire aims to reflect the clinicians' rationale and attitude towards the prescription of antipsychotic polypharmacy (APP) in the East Perth metropolitan area in Western Australia and investigate if other factors such as prescribing culture, compliance with treatment guidelines, or personal preference may be responsible for the psychiatrists' attitude towards APP.

Prescribers’ information

1. Clinician’s years of clinical experience:

2. On average, how many patients do you see per week?

Appendix Two: Research Questionnaire #2

**Table 4:** Research questionnaire #2

		Yes	No
1	Is your prescription rationale linked to medication cross-titration, failed clozapine trial and clozapine intolerance?		
2	Do the local/national guidelines impact your prescription practice?		
3	Does the patient’s risk for aggression impact your rationale for the prescription of APP		
4	Based on your expert opinion, does the justification for the prescription of APP correlates to its increased efficacy?		

## References

[1] Nakagami Y, Hayakawa K, Horinouchi T (2021). A call for a rational polypharmacy policy: international insights from psychiatrists. Psychiatry Investig.

[2] Correll CU, Shaikh L, Gallego JA, Nachbar J, Olshanskiy V, Kishimoto T, Kane JM (2011). Antipsychotic polypharmacy: a survey study of prescriber attitudes, knowledge and behavior. Schizophr Res.

[3] Armstrong KS, Temmingh H (2017). Prevalence of and factors associated with antipsychotic polypharmacy in patients with serious mental illness: Findings from a cross-sectional study in an upper-middle-income country. Braz J Psychiatry.

[4] Hjorth S (2021). The more, the merrier…? Antipsychotic polypharmacy treatment strategies in schizophrenia from a pharmacology perspective. Front Psychiatry.

[5] Lähteenvuo M, Tiihonen J (2021). Antipsychotic polypharmacy for the management of schizophrenia: evidence and recommendations. Drugs.

[6] Kim JJ, Pae CU, Han C, Bahk WM, Lee SJ, Patkar AA, Masand PS (2021). Exploring hidden issues in the use of antipsychotic polypharmacy in the treatment of schizophrenia. Clin Psychopharmacol Neurosci.

[7] Correll CU, Gallego JA (2012). Antipsychotic polypharmacy: a comprehensive evaluation of relevant correlates of a long-standing clinical practice. Psychiatr Clin North Am.

[8] Brett J, Daniels B, Karanges EA (2017). Psychotropic polypharmacy in Australia, 2006 to 2015: a descriptive cohort study. Br J Clin Pharmacol.

[9] James BO, Omoaregba JO, Raji SO (2017). Attitudes towards and rationale for antipsychotic polypharmacy among psychiatrists in Nigeria: Characteristics associated with high reported antipsychotic polypharmacy. Psychiatry Res.

[10] Fleischhacker WW, Uchida H (2014). Critical review of antipsychotic polypharmacy in the treatment of schizophrenia. Int J Neuropsychopharmacol.

[11] Kishimoto T, Watanabe K, Uchida H, Mimura M, Kane JM, Correll CU (2013). Antipsychotic polypharmacy: a Japanese survey of prescribers' attitudes and rationales. Psychiatry Res.

[12] Saldaña SN, Keeshin BR, Wehry AM, Blom TJ, Sorter MT, DelBello MP, Strawn JR (2014). Antipsychotic polypharmacy in children and adolescents at discharge from psychiatric hospitalization. Pharmacotherapy.

[13] Lin SK (2020). Antipsychotic polypharmacy: a dirty little secret or a fashion?. Int J Neuropsychopharmacol.

[14] Barbui C, Biancosino B, Esposito E, Marmai L, Donà S, Grassi L (2007). Factors associated with antipsychotic dosing in psychiatric inpatients: a prospective study. Int Clin Psychopharmacol.

[15] Abolmagd S, Aly El-Gabry D, Elkholy H, Aziz KA (2020). Tackling myths of common prescribing patterns in schizophrenia amongst Egyptian psychiatrists. Middle East Curr Psychiatry.

[16] K Khaja, M Al-Haddad, R Sequeira, A Al-Offi (2012). Antipsychotic and anticholinergic drug prescribing pattern in psychiatry: extent of evidence-based practice in Bahrain. Pharm Pharmacol.

[17] Divac N, Prostran M, Jakovcevski I, Cerovac N (2014). Second-generation antipsychotics and extrapyramidal adverse effects. Biomed Res Int.

[18] Tomlinson EJ, Rawson H, Manias E, Phillips NN, Darzins P, Hutchinson AM (2021). Factors associated with the decision to prescribe and administer antipsychotics for older people with delirium: a qualitative descriptive study. BMJ Open.

